# Medical Opinion and Movement

**Published:** 1911-01-07

**Authors:** 


### MEDICAL OPINION AND MOVEMENT.

### Cholesterine and Arterio-sclerosis.

At a meeting of the Académie des Sciences Dr. Lemoine read a paper on the relation between cholesterine and arterio-sclerosis. He draws attention to the fact that all substances having cholesterine as a base when administered by the mouth or injected under the skin raise the arterial tension, and the idea, therefore, suggested itself whether the development of arterio-sclerosis might not be affected by the action of these lipoids upon the walls of the arteries. On examination he finds that atheromatous plates are composed of cholesterine crystals with fatty matter and a calcareous sediment. In fact, the atheromatous plates consist of a mass of lipoids rich in cholesterine impregnating a deposit of calcium salts. In order to determine more definitely the relation of cholesterine to atheroma the author has estimated the proportion of cholesterine in the walls of arteries from patients of different types, and he found that whereas the aorta of a tuberculous subject showed only traces of cholesterine in its composition, those from patients with well-marked arterio-sclerosis contained a considerable amount of cholesterine amounting to 0.68 gramme and 0.70 gramme per 100 parts of aorta. Other subjects showed intermediate proportions. From these observations the author supposes that the deposits probably play an important rôle in the production of arterio-sclerosis.

### Perforated Gastric or Duodenal Ulcer.

Dr. Neumann devised about a year ago a new method of utilising the omentum in certain cases of perforated gastric or duodenal ulcer, in which the surrounding tissue is too friable to permit of suturing. His method consists in arranging the omentum in the form of a canal extending from the perforation to the external wound. In this canal he places a tube which just fits into the perforation and projects through the abdominal wound. In this way a drainage is established between the stomach or duodenum and the outside and completely shut off from the peritoneal cavity. Dr. Axhausen has just published a case in the *Deutsche Zeitschrift für Chirurgie* in which this method of procedure was successfully carried out. The patient was a man aged fifty-eight who had suffered for some time from symptoms of ulcer, and was admitted into hospital with acute peritonitis. Laparotomy was performed. A purulent effusion was found, especially in the upper part of the abdomen, and a perforation was discovered on the duodenal wall two fingers' breadth from the pylorus. The surface around was covered with false membrane and the wall was indurated and fixed. It was at once obvious that excision and suturing would be useless and the patient's condition was too serious for gastro-enterostomy. Dr. Axhausen therefore adopted the method already described, and a drain was also inserted under the liver and the rest of the wound closed. A large quantity of fluid was passed through the tube during the first forty-eight hours, but this rapidly diminished and the tube was withdrawn on the eighth day and the fistula closed soon afterwards. A month later marked signs of pyloric stenosis developed and gastro-enterostomy was performed under local anæsthesia. The patient recovered, and six months later was in good health.

### Diabetic Coma.

At a recent meeting of the Berlin Medical Society two interesting pieces of research were brought to light and reported upon, one dealing with the pathology of diabetic coma and the other with the glycogenic function of the liver. Dr. Ehrmann finds that a coma analogous to diabetic coma in the human organism can be produced in rabbits by the administration of sodium butyrate. The animal treated in this way becomes apathetic and unconscious, respiration is retarded so as to fall from eighty a minute to less than forty-five. The expirations are prolonged and the respiratory movements have a greater amplitude. The eyeball loses its tension just as in diabetic coma, and the urine contains diacetic acid and $\beta$-oxybutyric acid. There is a diminution of alkalinity and of carbonic acid in the blood. The author maintains that the diminution in carbonic acid is not responsible for the coma, as he has produced the same deficiency of carbonic acid in the blood by administering other substances such as sodium isobutyrate without inducing coma. The same results have also been obtained in dogs which, as the author points out, is the more satisfactory, seeing that canine metabolism is more closely analogous to human than is that of the rabbit. The "acidosis" theory of diabetic coma is based largely upon the fact that a condition in some way analogous to diabetic coma is produced in animals by the administration of acids, but sodium butyrate is neutral or slightly alkaline, and the condition produced appears to present a much closer similarity to diabetic coma. Dr. Ehrmann is still engaged in further research on the problem.

### Glycogenic Function of the Liver.

The experimental work on the glycogenic function of the liver was carried out by Dr. E. Frank. He took up the theory that this function of the liver is controlled by the varying composition of the pancreatic secretion in inorganic salts, certain inorganic combinations causing a retention and others a "mobilisation" of the carbohydrates in the liver. To determine this experimentally he set up an artificial circulation in the liver of the tortoise. If Ringer's solution with 2 per cent. of glucose is circulated through the liver the amount of glucose diminishes, but the liver content in glycogen increases. If, for instance, the amount of glycogen before the experiment was 3.3 per cent., this increased to 5.1 per cent. If, however, the potassium chloride is omitted from the Ringer's solution the hepatic glycogen diminishes. The same result is obtained if, instead of omitting the potassium chloride, the sodium bicarbonate is left out of the solution. Whether the omission of this salt determines this "mobilisation" of the hepatic glycogen, or whether the result is due to some interaction between the inorganic ions of the solution—*i.e.* between the sodium or potassium and the calcium—remains at present undetermined. It is interesting to note in this connection that recent physiological researches tend to emphasise the importance of the inorganic ions in all metabolic changes and cell activities.

### Cranial Osteo-periostitis in Infants.

Drs. Triboulet and Ribadeau-Dumas write in *Les Archives de Médecine des Enfants* on the symmetrical nodular osteo-periostitis of the flat bones of the skull met with in tuberculous infants. In view of the fact that it is difficult in a large number of cases to arrive at a definite diagnosis of tuberculous infection in the infant, the observations of the authors may prove helpful. They have noticed in several cases of acute tuberculosis occurring in nurslings, the appearance of true bosses in the frontal, parietal, and temporal regions, which are more or less tender to the touch. Histological and bacteriological examination of these showed them to be due to tuberculous lesions in the diploe and adjacent periosteum, occurring in the course of a severe or slight disturbance of digestion. The lesions are of two main varieties (1) a perforating sequestral form, and (2) one characterised by progressive infiltration. The first is the more common: bony caries ends in the production of a sequestrum with a sloping edge and larger on the side of the dura mater than on that of the pericranium: all around are fungoid growths and cold abscesses. When the subject is not cured spontaneously death follows from visceral tuberculosis or some intercurrent affection. In their chronic forms syphilis and tuberculosis of the cranial bones have much in common. Nevertheless the former disease more often gives rise to erosions, craniotabes, or to bosses which occur on the frontal and parietal regions. In the syphilitic lesion there is a transparent glassy effusion and not true pus as in tubercle.

### Infantile Scurvy.

Dr. Comby has contributed a paper on infantile scurvy to a recent number of the *Archives de Médecine des Enfants* in which he states that he has observed fifteen cases of this disease during the last twelve years. Of the fifteen babies observed by the author, only one had been breast-fed, and this one had become scorbutic only after a prolonged diet of starchy foods and boiled milk. In three cases the children had been fed on commercial sterilised milk and paps made with the same fluid. In these a cure was effected by stopping the milk, but continuing the paps. "Fixed sterilised milk" also called "homogeneous milk" accounted for four, and "oxygenised milk" or "nectar" for one case. Among the most scorbutigenous milks must be placed "humanised milk" prepared according to Gaertner's formula, since this fluid gave rise to six of the author's cases. He has never seen a case of scurvy resulting from breast-feeding, nor from artificial feeding carried out with milk sterilised at home. In so far as fresh food, fruit juice, soups of vegetables, bread-crusts, etc., are added to these preserved milks, so are the dangers of scurvy mitigated in almost all cases. Diagnosis in the author's series was easy owing to the presence of purpura, œdema, grave anæmia, gingival hæmorrhages with fungosities, but especially of the painful pseudo-paraplegia which when present always is a characteristic and arresting symptom. The children were cured quickly by the administration of fresh milk to which a few spoonfuls of orange-juice were added.

### Tumours of the Third Ventricle.

The symptom complex characteristic of growths involving the third ventricle of the brain has been worked out by Dr. T. H. Weisenberg, who reports cases in *Brain* and analyses exhaustively the signs upon which it is possible to make the diagnosis. These tumours, whether growing from the ependymal walls or from the choroid plexus, are not very common; but a good many cases have been reported of various different kinds. The nature of the growth has no direct bearing upon the symptoms; they have a tendency to spread along the line of flow of the cerebrospinal fluid, that is along the aqueduct of Sylvius into the fourth ventricle. Internal hydrocephalus is nearly always caused, as might be expected. The recognition of these growths depends entirely on the pressure symptoms excited in surrounding structures. Thus tumours of moderate size, limited to the third ventricle, cause the symptoms of internal hydrocephalus, with the addition of paresis of the limbs of one or both sides, and sometimes of signs referable to the optic thalamus. Tumours which are so situated as to obstruct the foramina of Munro cause dilatation of the lateral ventricles; their position can sometimes be changed by movements of the head, and variations in the symptoms may be thus produced. Then, again, tumours which attack the parts surrounding the aqueduct of Sylvius, either by direct pressure or after extension into the aqueduct by growth of the tumour ventrally, cause paralysis of associated ocular movement and of convergence upwards, and (less commonly) to either side and downwards; ataxia of cerebellar type, as shown in the gait; ptosis of the upper lids and protrusion of one or both eyes, generally, large pupils with impaired reactions; paresis of the limbs of one or both sides, with increased, normal, or diminished reflexes; and, lastly, the general symptoms of cerebral tumour, headache, choked disc, nausea, vomiting, vertigo. Lesions of the third ventricle do not cause specific mental symptoms, though these may occur from the resulting hydrocephalus, which compresses the cortex.

### Treatment of Arthritis Deformans.

In the *American Journal of the Medical Sciences* Skinner sums up his views of the therapeutics of this troublesome complaint. He lays stress on rest in bed for at least ten hours out of every twenty-four, and on a diet as generous as can be assimilated without upsetting the digestion. He gives $\frac{1}{30}$ to $\frac{1}{40}$ grain of strychnine sulphate, and two to three grains of ferrous iodide three times daily half an hour before meals; and in emaciated patients one to four drachms of cod-liver oil after meals. If constipation is present, he treats it with a dose of some approved mineral aperient before breakfast every other day. Then he orders also a body dry hot-air treatment two or three times a week, and central galvanisation once or twice a week. The general application of mechanical vibratory stimulation two or three times a week is also recommended, as also is a hot and cold douche to the spine two or three times weekly. Static and high-frequency currents he also uses in certain cases. For the relief of pain, besides the measures already enumerated, hot-water bags and hot fomentations are of use; Bier's hyperæmia has proved disappointing in the author's practice. As drugs for pain he finds useful tincture of iodine externally, and a mixture of chloral camphor and aconite internally. Opium is inadvisable, and the coal-tar hypnotics also do not find favour. Salicylates and the tinctures of hyoscyamus and gelsemium are mentioned with approval. The omission of guaiacum and iodides from this list will strike British practitioners as a remarkable feature.

### Skin Disinfection.

THE revival of the iodine method of skin disinfection by Grossich in 1908 seems to have led many surgeons to experiment for themselves with substances which had rather fallen out of vogue a few years ago. Selter advocates, in the *Deutsch. Med. Wochensch.* an alcoholic paste for the sterilisation of the surgeon's hands. This is a mixture of 86 parts of absolute alcohol and 14 parts of soap. About five grams of this (75 grains) is rubbed well in for about five minutes. Zetti, on the other hand, advocates in the *Wiener Klin. Wochensch.* the use of petroleum followed by benzine. The area to be operated on is shaved, if necessary, on the preceding day; but no bathing or other preliminary antiseptic treatment is allowed. A mop of gauze or wool is then soaked in petroleum, and the skin is rubbed with it for one and a half minutes. Should the swab become soiled, the process is carried out again. Then a swab soaked in benzine is similarly used for half a minute. The author has tested this routine in seven hundred cases, and is convinced that it is efficacious. He also notes an absence of dermatitis or any other evidence of irritation. Yet a further advantage claimed is that the skin is left slightly oily, and that in this state blood does not adhere to it, and this not unusual source of wound infection is therefore not to be feared.

### Elimination of Hexamethylenamine.

THIS drug is frequently sold under the protected trade name of urotropine, when it costs, of course, much more than if the product be bought by the scientific name given to it in accordance with its chemical formula. It is, as is well known, used chiefly as a urinary antiseptic; and it is supposed that this action is due to the liberation of formaldehyde by its decomposition. Dr. Barton, in a communication to the American Therapeutic Society, published in the *Monthly Cyclopædia and Medical Bulletin*, claims that the drug is also to some extent eliminated by the mucous membrane of the middle ear and accessory nasal sinuses. He finds that hexamethylenamine is of service in acute suppurative otitis media; all the cases (seven in number) in which he tried it were rapidly cured. Chronic cases are ameliorated, but no absolute cure has yet been ascertained. Upon *a priori* grounds he assumes that this compound may prove a valuable prophylactic in those diseases commonly complicated by otitis media; and possibly also prior to surgical operations upon the middle ear, mastoid, or nose. The suggestion is one quite worth testing, and any medical officer of a fever hospital who will test it on a large series of consecutive cases of scarlet fever, for instance, will be doing service to the community.

### Burns in Children.

DR. JUDD contributes to the *Postgraduate* an account of the treatment he adopts for burns in children. A few years ago somewhat heroic measures were preached to combat these serious lesions; scrubbing the burnt surface with nailbrushes steeped in biniodide (under anæsthesia) was one of the processes advocated, but it never " caught on " among those who have to deal with these cases. The author, however, is in favour of cleansing the burnt surface under gas, or light chloroform or ether anæsthesia; though he does not adopt the severe measures just mentioned. When bullæ are present, they are snipped away, and all dirt and foreign material is removed with sterile swabs and green soap. Then the whole raw surface is irrigated with normal saline. In very dirty cases the surgeon takes a little commercial chloride of lime, adds a crystal of bicarbonate of soda, and makes a paste with a little water. With this the part is cleansed, care being taken to wash away thoroughly all the lime by repeated douches of warm saline afterwards. When this stage of cleansing has been completed, in some cases weak boric acid ointment is used as a dressing and hot saline baths are given once or twice a day. The most ideal method, however, is said to be to spread cargile membrane over the surface of the wound. This is the sterilised peritoneum of the ox, cut into strips. The membrane applies itself closely to the surface, and relieves pain as nothing else can. Over the cargile a moist weak antiseptic dressing is used, changed as required. Discharge passes freely through the cargile, which therefore need not be changed.

### Treatment of Crushed Limbs.

IN *La Semana Medica*, Dr. Natal Lopez Cross gives an account of the method usually employed at the San-Roque Hospital, Buenos Ayres, in dealing with cases in which hurried amputation is necessary. The old-fashioned circular amputation is performed, but no attempt is made to form flaps, skin, muscles, and bone being cut through at the same level usually about three centimetres above the crushed area. Bleeding vessels are then tied, and the stump dressed with an antiseptic dressing. Some three weeks later, when suppuration has ceased, the wound is healthy, and all danger of infection is past, a more scientific amputation at the site of election can be carried out with safety.

